# Study on MoO**_3_**/SBA-16 catalyzed transesterification to synthesize diphenyl carbonate

**DOI:** 10.55730/1300-0527.3492

**Published:** 2022-08-10

**Authors:** Li YING, He SHUNWU

**Affiliations:** 1 School of Chemical Engineering, Chongqing University of Technology, Chongqing, China; 2 School of Chemistry and Chemical Engineering, Guizhou University, Guiyang, Guizhou, China

**Keywords:** MoO_3_/SBA-16, transesterification, dimethyl carbonate, phenyl acetate, diphenyl carbonate

## Abstract

In this paper, the mesoporous molecular sieve SBA-16 with good dispersion was prepared by hydrothermal method. Under the condition of Si/Mo= 1.0, MoO_3_/SBA-16 catalysts were prepared by iso-dipping method at 550 °C, 650 °C and 750 °C calcination temperature, which for the synthesis of diphenyl carbonate (DPC) from dimethyl carbonate (DMC) and phenyl acetate (PA) was used, and the uncalcined MoO_3_/SBA-16 was used as a reference. XRD, TG-DTA, XPS, ICP-MS, FT-IR, BET, SEM and TEM characterization. The effects of calcination temperature and reaction conditions on the catalytic performance of MoO_3_/SBA-16 in transesterification of DMC and PA were investigated. The results show that the MoO_3_/SBA-16 catalyst prepared at 550 °C has the best catalytic performance in the reaction of DMC and PA ester exchange, and the catalyst has the best catalytic performance in the appropriate reaction strip (n(PA) = 1.0 mol; n (PA)/n (DMC) = 2.0; m (MoO_3_/SBA-16) = 6.0g; t = 5.0 h; T = 180 °C), the DMC conversion is 78.5%, and the selectivity of methyl phenyl carbonate (MPC) and DPC is 51.1% and 46.5%, respectively. After five cycles, the catalyst activity decreased and the conversion of DMC decreased from 78.5% to 15.8%. However, its crystal structure remained unchanged. The used catalyst can be easily regenerated by calcination in air at 550 °C for 5 h. The catalytic performance of the regenerated catalyst was almost as high as that of the fresh one.

## 1. Introduction

At present, more and more people pay close attention to green environmental protection issues, especially in the industrial field of engineering plastics development. Polycarbonate (PCs) is one of the best engineering plastics, and the optimization of its production process still poses great challenges to the researchers. The traditional production process is facing elimination because phosgene is highly toxic and causes serious environmental safety problems. Diphenyl carbonate (DPC) replaces phosgene to synthesize polycarbonate by transesterification, and the green production technology of DPC by nonphosgene method has become a research hotspot in recent years [1–5]. Among them, the technology of synthesizing DPC with dimethyl carbonate (DMC) and phenyl acetate (PA) as raw materials has a good prospect for realizing cleaner production of DPC because of its favorable thermodynamics and meeting the requirements of “atomic economy”. The development of efficient catalyst system is one of the key technologies for synthesizing DPC from DMC and PA.

Mesoporous molecular sieve materials have large specific surface area, relatively large pore size and regular pore structure, and can treat large molecules or groups, therefore they are good shape-selective catalysts [6]. SBA-16 molecular sieve is a typical one among many molecular sieves. It has a large cage cubic symmetric structure with uniform size, thick pore wall, high specific surface area and thermal stability, especially the connectivity of its three-dimensional pore channels is more conducive to material transport and diffusion of reaction molecules. Therefore, it has a good application prospect in catalysis, adsorption, separation and biomolecule immobilization [7–13]. However, a single SBA-16 molecular sieve has only a few active centers, and it is easy to deactivate when it is used as a catalyst alone. However, in recent years, some researchers have found that the oxidation active center of SBA-16 can be increased and the catalytic performance can be improved by adding metal. In Akti’s study [14], copper and iron SBA-16 catalysts were synthesized by hydrothermal method, and the catalysts were modified with potassium. The results show that the metal exists in SBA-16 structure in the form of nanoparticles, which increases the peak strength of Bronsted and Bronsted Lewis acid centers of SBA-16, and reduces the specific surface area of SBA-16 molecular sieve, but the catalyst has excellent performance. At 350 °C, the modified catalyst shows high conversion rate and acetaldehyde selectivity can reach 81%. Feliczak-Guzik et al. [15] modified SBA-16 synthesized by niobium, ruthenium, palladium and platinum. The obtained material is used as a catalytic system in the process of deoxidization by hydrogenation of phenol. When the reaction temperature is 90–130 °C and the hydrogen pressure is 2.5–6 MPa, the conversion rate of phenol reaches 100%. Gholizadeh et al. [16] used SBA-16 as carrier and TiO_2_ as active component to synthesize a semiconductor compound by one-pot method. The analysis results show that the active center of metal modified SBA-16 is increased, and under the optimum conditions, TiO_2_/SBA-16 photocatalyst can improve the phenol conversion rate. Bian et al. [17] synthesized a series of Ni/SBA-16 and Ni Mo/SBA-16 catalysts, which were used for catalytic synthesis of natural gas (SNG) in fluidized bed reactor. When MoO_3_ is added to the Ni/SBA-16 catalyst, the new catalyst shows a larger metal surface area and better thermal stability. There is a strong interaction force between the metal Ni and the support SBA-16. The Mo-Ni/SBA-16 (1 wt%) catalyst has the highest reactivity under the reaction conditions of 350 °C, 0.1 MPa, and 15,000 mL/g/h, with a CO conversion rate of 100% and a CH_4_ selectivity of 97%. The activity of Mo-Ni/SBA-16 (1 wt%) catalyst remained stable after being calcined at 700 °C for 100 h in reactive gas atmosphere. Similar research [18], modified SBA-16 molecular sieve with three-dimensional mesoporous CeO_X_, catalyzed the oxidation of bisphenol A by the prepared catalyst, which effectively removed the ozonation of bisphenol A and increased the conversion rate of bisphenol A. On the one hand, the CeO_X_/SBA-16 mesoporous structured materials presented large surface area and uniform pore distribution, which was conducive to the adsorption of transformation byproducts (TBPs) and then, the mass transfer. On the other hand, CeO_X_/SBA-16 could enhance the ozone utilization efficiency and meanwhile facilitate the formation of OH, the main reactive oxygen species. This strategy will be beneficial to the design and construction of mesoporous materials, so as to catalytically eliminate the harm to the environment, and provide a feasible development prospect for mesoporous materials in the process of production.

High purity and dispersion of SBA-16 molecular sieves were prepared by hydrothermal method and physically mixed with MoO_3_. MoO_3_/SBA-16-550, MoO_3_/SBA-16-650, and MoO_3_/SBA-16-750 were prepared at calcination temperatures of 550 °C, 650 °C, and 750 °C, respectively, with high efficiency. Then, the uncalcined MoO_3_/SBA-16-0 catalysts were used as reference materials to further demonstrate that the calcination temperature is an important influencing factor for catalyst synthesis. The catalyst performance was analyzed in combination with microstructural and morphological characteristics. In addition, the effects of calcination temperature and reaction conditions on the catalytic performance of the catalysts were investigated.

## 2. Experimental

### 2.1. Sample preparation

According to the literature report [19,20], SBA-16 molecular sieve was prepared by hydrothermal method. Generally, SBA-16 molecular sieve was synthesized hydrothermally with TEOS as silicon source and F127 triblock polymer (EO_106_PO_70_EO_106_) as template under strong acidic conditions. The molar ratio of reactants is TEOS: F127: HCl: H_2_O = 1: 0.004: 0.75: 88. The resultant solution is stirred in a water bath at 40 °C for 24 h, and then hydrothermal crystallized in an oven at 100 °C for 48 h. After being cooled to room temperature, these samples were filtered, washed with deionized water, dried in an oven at 100 °C for 12 h, and baked in a tube furnace at 550 °C for 6 h at a heating rate of 2 °C/min to obtain SBA-16. The next step is grinding SBA-16 into particles with the particle size of 20–40 meshes, and preparing for the subsequent operation. Then, MoO_3_/SBA-16 catalysts with different calcination temperatures were prepared by impregnation method with mesoporous molecular sieve SBA-16 as carrier, ammonium molybdate ((NH_4_)_6_Mo_7_O_24_·4H_2_O) as Mo source, and Si/Mo = 1.0. A certain amount of ground SBA-16 was weighed and soaked in ammonium molybdate aqueous solution with a certain concentration, and then dried in an oven at 100 °C for 24 h, so as to prepare a primary sample of MoO_3_/SBA-16-0. Then, MoO_3_/SBA-16 catalysts, named MoO_3_/SBA-16-550, MoO_3_/SBA-16-650 and MoO_3_/SBA-16, were obtained by calcination in a tube furnace at 550 °C, 650 °C and 750 °C, respectively.

### 2.2. Characterization methods

The small-angle X-ray diffraction of the sample was determined by Bruker D8 Advance X-ray diffractometer (40kV, 200mA), with Cu target Ka line (0.154056 nm) as the incident light source, Ni filter, scanning speed of 0.5°/min, scanning range of 0.5–5°, tube voltage of 40 kV and tube current of 40.

The wide-angle X-ray diffraction pattern of the sample was recorded on D/max 2500 X-ray diffractometer, which operated at 40 kV voltage and 40 mA current, with Cu target Kα radiation (λ = 0.154056 nm), scanning speed of 2°/min and scanning range of 10–80°.

The samples were analyzed by thermogravimetric differential thermal analysis (TG-DTA, STA2500). All samples were heated under air atmosphere at a constant rate of 10 °C/min at a constant rate from room temperature to 800 °C, and TG and DTA curves were recorded simultaneously.

X-ray photoelectron spectroscopy (XPS) was performed on a Thermo K-Alpha + using a monochromatic AlKαX-ray source (1486.6eV).

ICP-MS was used to determine the content of Mo elements in the samples, which were detected by an inductively coupled plasma mass spectrometer (Perkin Elmer Nexion 300).

The specific surface area (BET) of the catalyst was measured by an automatic physical and chemical adsorption tester (Micromeritics Tristar 3000, USA). With high purity N_2_ as adsorbate, the sample was degassed in vacuum at 300 °C for 4 h, and then the N_2_ adsorption-desorption isotherm of the sample was measured at 77 K. The specific surface of the sample was calculated according to BET equation in the range of P/P_0_= 0.05–0.3, and the pore distribution was calculated by using the equivalent cylindrical model BJH.

The skeleton structure of the samples was analyzed by a Nicolet IS50 FTIR spectroscope using KBr pellets (KBr-to-sample mass ratio = 1:500).

The morphology of the samples was observed on ZEISS IGMA+X-Max 20 by Phenom G5 scanning electron microscope (SEM) under the working voltage of 15kV. Transmission electron microscope (TEM) was carried out on the samples at an operating voltage of 200kV.

### 2.3. Reaction performance test

The reaction performance of MoO_3_/SBA-16-0, MoO_3_/SBA-16-550, MoO_3_/SBA-16-650, and MoO_3_/SBA-16-750 catalysts for diphenyl carbonate synthesis was evaluated in a high-pressure reactor (250mL). The reaction was carried out with 40–60 mesh catalyst, and the molar ratio of dimethyl carbonate (DMC) to phenyl acetate (PA) was 1:2. The raw materials and catalyst were added into the reaction kettle according to the metering ratio, and the gas tightness test was carried out with high purity nitrogen after sealing. Then the reaction is carried out at the ambient pressure and temperature of 180 °C. The agilent-7820 online gas chromatograph equipped with flame ionization detector and HP-5 capillary column (30 m× 0.53 mm× 0.25 μm) was used to test the products online. In the reaction, water generated by esterification is separated by water ring vacuum pump in time. After the reaction is finished, when the temperature is lowered to room temperature, the materials in the kettle are discharged, and the products are obtained by filtration. The mixture of DMC and PA ester exchange reaction was first distilled under reduced pressure (vacuum of 1999.8 pa), and the first fraction was collected within the fraction temperature of 60–80 °C for the unreacted PA, and the second fraction was collected within 80–130 °C for the mixture of DPC and MPC, and the catalyst was left in the distillation kettle. The second fraction is dissolved in anhydrous ethanol for recrystallization, and pure DPC is obtained. The reaction mechanism is as follows:


(1)

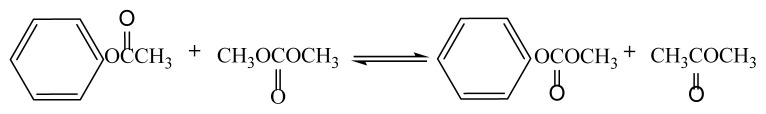



(2)

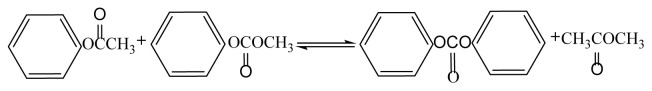



(3)

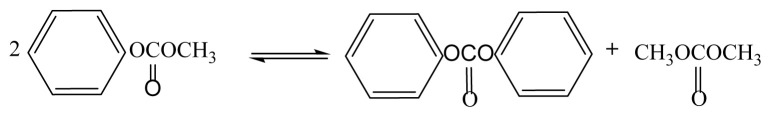


Diphenyl carbonate (DPC) is synthesized from dimethyl carbonate (DMC) and phenyl acetate (PA) in a two-step conversion. In the MoO_3_/SBA-16 catalyst system, the intermediate methyl phenyl carbonate (MPC) is firstly formed by the reaction of dimethyl carbonate with phenyl acetate as in Eq. (1), the intermediate MPC is not stable. On the one hand, it can be reacted with PA to obtain the target product DPC as in Eq. (2), on the other hand, it can also be disproportionated by itself to generate DPC according to Eq. (3).

### 2.4. Analysis procedure

The performance was tested by gas chromatography (Angilent7820). DMC conversion *CR*(DMC), MPC selectivity *S*(MPC), and DPC selectivity *S*(DPC) were calculated using the following equations:


(4) CR(DMC)=fi.Ai∑(fi.Ai)

(5) $$S_{\left(MPC\right)}=\frac{n_{\left(MPC\right)}}{n_{0(MPC)}}\times 100\%$$

(6) $$S_{\left(DPC\right)}=\frac{n_{\left(DPC\right)}}{n_{0(DPC)}}\times 100\%,$$

where *A_i_*, *f_i_*, n(MPC), n_0_(MPC), n(DPC), n_0_(DPC) denote the peak area of the component (mAU*min), the correction factor, the amount of MPC substance actually obtained (mol), the amount of MPC substance theoretically obtained for the reaction system (mol), the amount of DPC substance actually obtained (mol), and the amount of DPC substance theoretically obtained for the reaction system (mol), respectively.

## 3. Results and discussion

### 3.1. The structure performance of the catalyst

**Figure 1:**
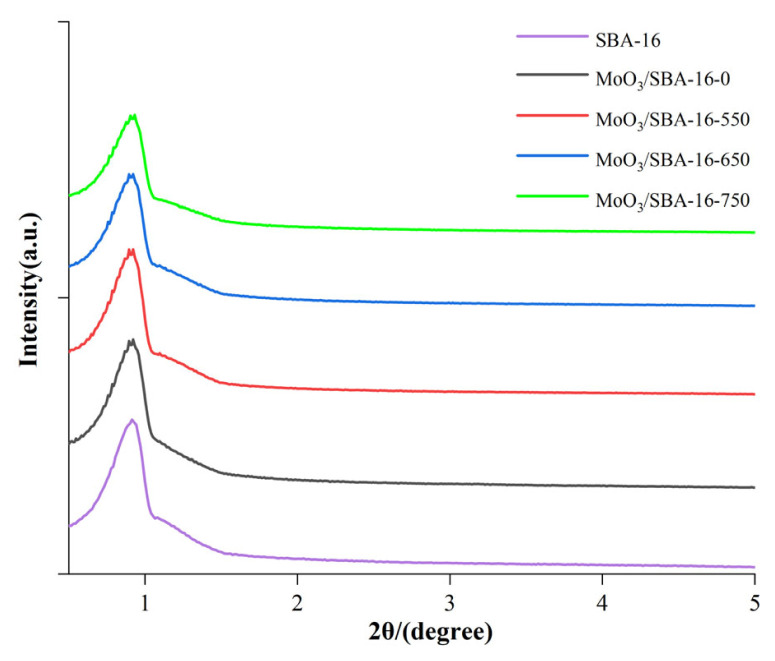
Low-angle XRD patterns of MoO_3_/SBA-16 catalysts at different calcination temperatures.

Figure 1 is a low-angle (0.5–5°) X-ray powder diffraction diagram of SBA-16 mesoporous molecular sieve and MoO_3_/SBA-16 catalyst with different calcination temperatures. From the diagram, it can be observed that SBA-16 has a characteristic diffraction peak of (110) crystal plane with cubic core Im3m structure around 2θ = 0.72°, which indicates that the prepared SBA-16 molecular sieve has a good long-range ordered mesoporous structure [21,22]. The characteristic diffraction peaks of the samples still exist after Mo is added into the molecular sieve framework, which indicates that the cubic ordered structure of the mesoporous materials still remains after Mo is added. At the same time, compared with SBA-16, MoO_3_/SBA-16 with different calcination temperatures has no change in the crystal plane structure of the characteristic diffraction peaks, which indicates that their ordered structure has not changed significantly after Mo source impregnation. Interestingly, with the increase of calcination temperature, the position of the characteristic diffraction peak of (110) crystal plane of MoO_3_/SBA-16 slightly shifts to a high angle and the intensity decreases, which is caused by MoO_3_ entering into the pores of SBA-16 molecular sieve or part of MoO_3_ accumulating in the pores.

**Figure 2:**
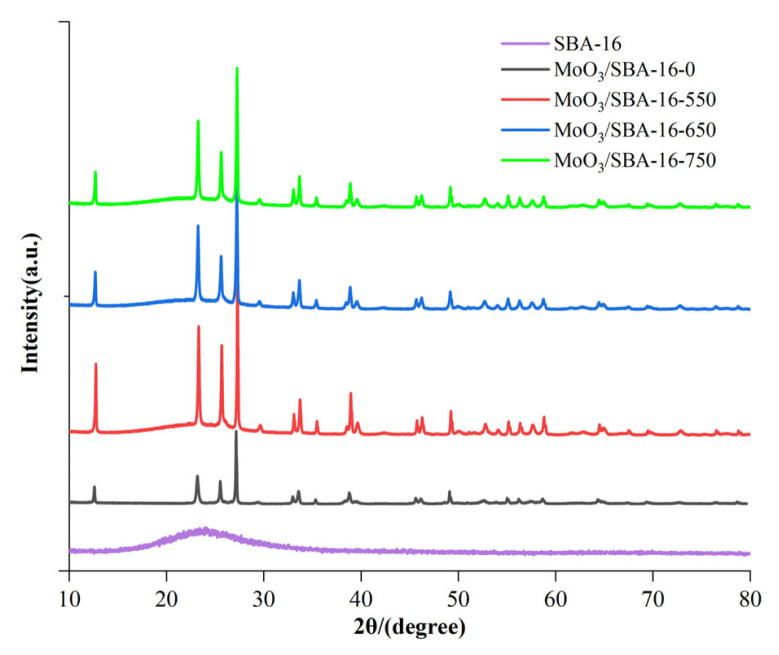
High-angle XRD patterns of MoO_3_/SBA-16 catalysts at different calcination temperatures.

Figure 2 is a high angle (10–80^°^) X-ray powder diffraction diagram of SBA-16 mesoporous molecular sieve and MoO_3_/SBA-16 catalyst with different calcination temperatures. It can be seen from Figure 1 that both SBA-16 and MoO_3_/SBA-16 samples have a diffraction peak attributed to amorphous SiO_2_ at about 2θ of 23.6°. In addition, the characteristic diffraction peaks of MoO_3_/SBA-16 belonging to crystalline MoO_3_ appeared at different calcination temperatures, in which 2θ = 12.8, 23.6, 25.9, 27.7, 39.2 corresponds (020), (110), and 39.2 corresponding to the standard PDF card (MoO_3_, JCPDS05-0508). This indicates that MoO_3_ in MoO_3_/SBA-16 material is either highly dispersed on the surface of SBA-16 [23], or the oxide particle size formed is not smaller than the detection line of XRD (5 nm). However, MoO_3_ characteristic diffraction peak intensity of MoO_3_/SBA-16-0 is weak, while MoO_3_/SBA-16-550 shows strong MoO_3_ characteristic diffraction peak. With the increase of temperature, the diffraction peak intensity of active component MoO_3_ begins to decrease.

**Figure 3:**
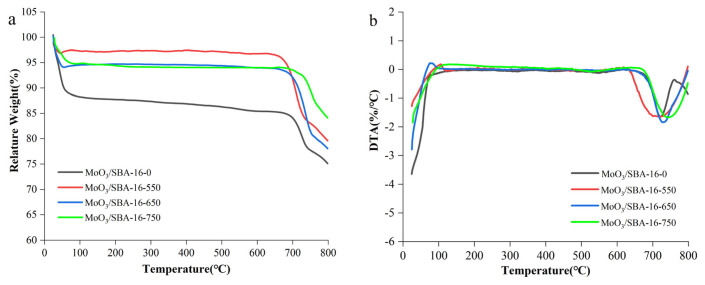
TG/DTA curves of MoO_3_/SBA-16 catalysts. (a) TG curve; (b) DTG curve.

Figure 3 shows the TG/DTA curves of MoO_3_/SBA-16 catalyst. From Figure 3a, it can be seen that significant mass weight loss occurs from 20–60 °C to 650–800 °C. The weight loss from 20–60°C is due to the volatilization of adsorbed water in the MoO_3_/SBA-16 catalyst, while the weight loss in the 650–800 °C interval is mainly attributed to the sublimation property of MoO_3_ at higher temperatures [24]. The corresponding DTA curve Figure 3b shows that the catalyst has no obvious absorption peak and no obvious mass change on the TG curve in the 110–600 °C interval, which is mainly attributed to the spontaneous dispersion of MoO_3_ on the surface of SBA-16 when the MoO_3_ and the high specific surface carrier SBA-16 are mixed and calcined at different temperatures, and the chemical bonding between the dispersed MoO_3_ and SBA-16 chemical bonding occurred, and MoO_3_ was firmly bound to SBA-16. The bonding between MoO_3_ and SBA-16 is strong. With the increase of temperature, MoO_3_ has the property of sublimation at higher temperature. This corresponds to the tendency of weight loss in the presence of TG. In a comprehensive analysis, the degree of weight loss of the catalysts changed with the increase of calcination temperature, and the weight loss of MoO_3_/SBA-16-0, MoO_3_/SBA-16-550, MoO_3_/SBA-16-650, and MoO_3_/SBA-16-750 were 9.66%, 16.94%, 15.41%, and 9.68%, respectively, which indicated that the MoO_3_ /SBA-16-550 catalysts with high MoO_3_ loading.

**Figure 4:**
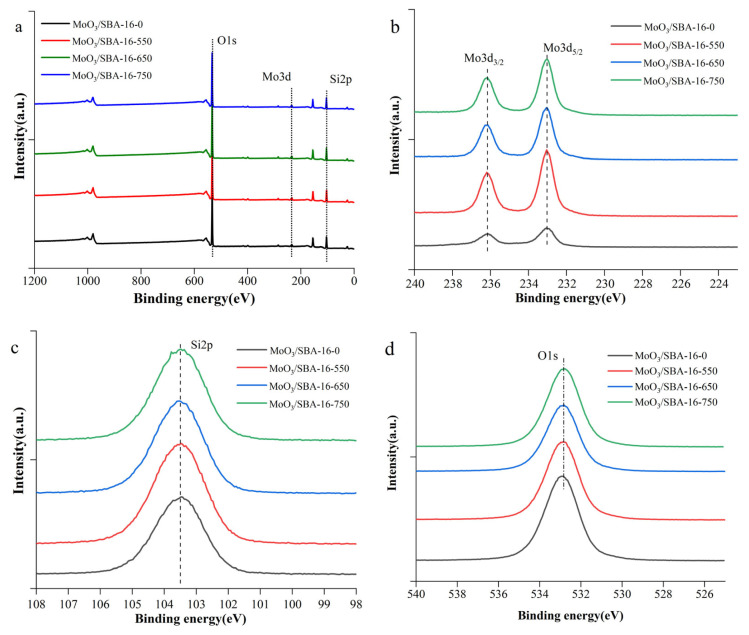
XPS diagrams of MoO_3_/SBA-16 at different calcination temperatures. a: The full spectrum; b: Mo3d photoelectron energy diagram; c: Si2p photoelectron energy diagram; d: O1s photoelectron energy diagram.

In order to investigate the elemental composition of the catalysts, XPS tests were performed on the catalysts with different roasting temperatures. As can be seen from Figure 4a, the catalysts all contain absorption peaks of three elements, Mo, O and Si. As shown in Figures 4a and 4b, in the Mo3d spectra of the catalysts, the main peaks at 232.9(+ 0.2) eV and 236.1(+ 0.2) eV were attributed to the MoO_3_ main peaks in the Mo3d5/2 and Mo3d3/2 orbitals, respectively, and the intensity of the peak signals changed after different calcination temperature treatments [25]. According to the fitting of catalyst Si2p in Figure 4c, the main peak at 103.4eV is attributed to the SiO_2_ main peak of SBA-16. As shown in Figure 4d, the fitted analysis of all catalyst surface O1s showed that the surface oxygen species were mainly lattice oxygen (531.61–534.12 eV).

**Figure 5:**
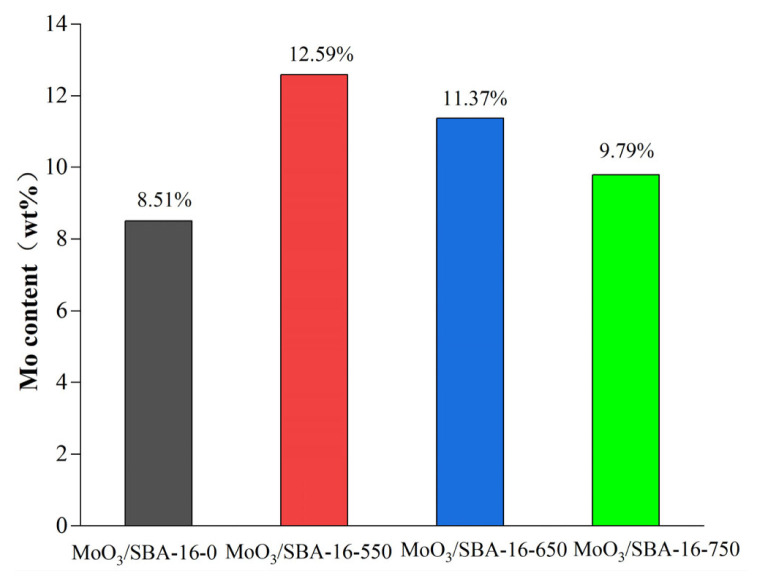
ICP-MS plot of Mo content of MoO_3_/SBA-16.

The Mo content of the catalyst was determined by ICP-MS. According to Figure 5, it is known that the Mo content of the catalyst was maximum at the calcination temperature of 550 °C, reaching 12.59%. The amount of Mo decreased gradually with the increase of calcination temperature, and the amount of Mo of the catalyst at 750 °C was similar to that of the catalyst without calcination. This indicates that the calcination temperature can change the amount of Mo of the catalyst.

The specific surface area, total pore volume and average pore diameter of SBA-16 molecular sieve and MoO_3_/SBA-16 catalyst at different temperatures are shown in Table 1. According to the data in Table 1, SBA-16 synthesized by hydrothermal method has good mesoporous structure, large specific surface area and pore volume, and the mesoporous area is as high as 807.6134 cm^2^ g^−1^. The modified MoO_3_/SBA-16 catalyst has obvious mesoporous structure of SBA-16 molecular sieve, but the mesoporous area is greatly reduced, ranging from 110 cm^2^ g^−1^ to 300 cm^2^ g^−1^. Interestingly, the catalysts at different calcination temperatures of Si/Mo = 25 still show microporous properties. The uncalcined MoO_3_/SBA-16-0 catalyst has the smallest specific surface area and mesoporous volume, and the particle polymerization degree is higher and the dispersion degree is smaller. It is worth mentioning that MoO_3_/SBA-16-550 catalyst prepared at 550 °C has a larger surface area than MoO_3_/SBA-16-0, MoO_3_/SBA-16-650 and MoO_3_/SBA-16-750. Compared with MoO_3_/SBA-16-550 catalyst, the specific surface area and mesoporous volume of MoO_3_/SBA-16-650 catalyst decreased, but compared with MoO_3_/SBA-16-0 catalyst, the specific surface area increased and the mesoporous volume decreased, accounting for 48.82%.When the calcination temperature reaches 750 °C, the specific surface area and mesoporous pore volume of the prepared MoO_3_/SBA-16-750 catalyst decrease correspondingly, which is similar to that of MoO_3_/SBA-16-0 after calcination, while the pore volume decreases to 27.87%. The evolution may be due to the addition of Mo content and the increase of temperature, which makes the special structure between Si-O-Mo inhibit the phase transition of MoO_3_. On the one hand, the stronger temperature effect is not enough to make MoO_3_’s weaker van der Waals force effect produce better performance and efficacy. On the other hand, when MoO_3_ is blended with SBA-16, part of MoO_3_ directly enters SBA-16 channels, blocking the channels, which leads to too high temperature, which reduces the specific surface area and mesoporous volume of MoO_3_/SBA-16-750 materials. Therefore, MoO_3_/SBA-16 needs proper temperature range and conditions to increase more active sites to improve its activity performance. It can be seen that the calcination temperature of 550 °C makes the phase surface of MoO_3_ and the phase surface of SBA-16 combine to create a special structure effect, resulting in the effect of increasing the active center, so that the surface of SBA-16 can form oxygen vacancies at a suitable temperature and activate Active sites improve the performance of the material.

**Figure 6:**
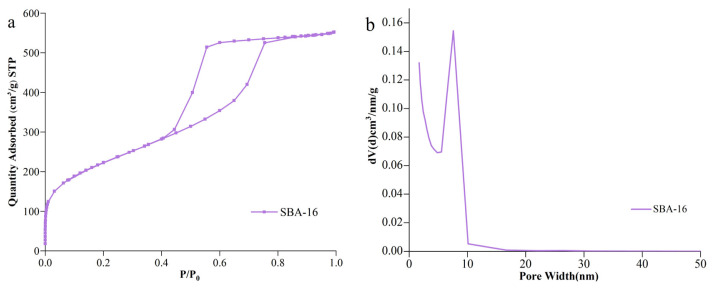
BET images of SBA-16 molecular sieve. (a) Isothermal adsorption line; (b) BJH hole size distribution diagram.

Figure 6 shows the BET analysis of SBA-16 molecular sieve. It can be seen from N_2_ adsorption/desorption isotherm of SBA-16 molecular sieve (Figure 6a) that SBA-16 prepared by hydrothermal method at 550 °C belong to typical type IV isothermal adsorption line [26]. When the relative pressure P/P_0_ < 0.1, the adsorption and desorption isotherms of the prepared SBA-16 mesoporous molecular sieve are closed, forming a typical type IV adsorption curve H2 hysteresis loop. When the relative pressure is P/P_0_ = 0.45–0.90, there is a clear hysteresis loop, which indicates that SBA-16 mesoporous molecular sieve has a large number of mesoporous structures. Barrett–Joyner–Halenda (BJH) pore size distribution (Figure 6b) data show that the prepared SBA-16 mesoporous molecular sieve has only one mesoporous pore size distribution, and the distribution is wide, with a pore size of 7.52 nm.

**Figure 7:**
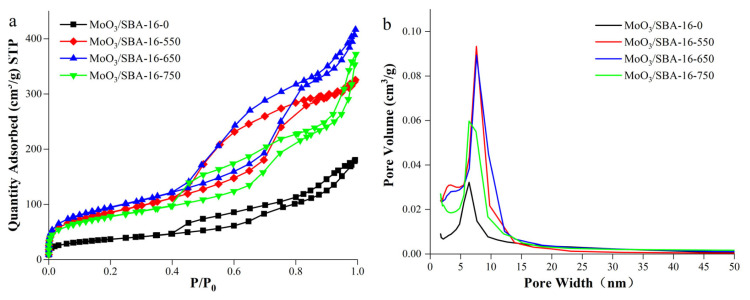
BET images of MoO_3_/SBA-16 catalyst at different calcination temperatures. a: Isothermal adsorption line; b: BJH hole size distribution diagram.

Figure 7 shows the BET analysis of the BET analysis of MoO_3_/SBA-16 catalysts with different calcination temperatures. It can be seen from N_2_ adsorption/desorption isotherm of MoO_3_/SBA-16 catalyst at different calcination temperatures (Figure 7a) that according to the classification of the international union of pure and applied chemistry, all samples show typical type IV adsorption curve H2 hysteresis loop, indicating that MoO_3_/SBA-16 catalyst is a typical three-dimensional cubic cage-like pore structure [27]. It can be seen from Barrett–Joyner–Halenda (BJH) pore size distribution (Figure 7b) that the pore size distribution of all catalysts is in a narrow range of 0.4–97 nm, which indicates that the pore size distribution of MoO_3_/SBA-16 catalyst is uniform. However, with the gradual increase of calcination temperature, the average pore size of MoO_3_/SBA-16 catalyst decreased from 12.58 nm to 6.37 nm, indicating that excessive molybdenum species may accumulate on the inner surface of the material. This is consistent with the analysis results of XRD.

**Figure 8:**
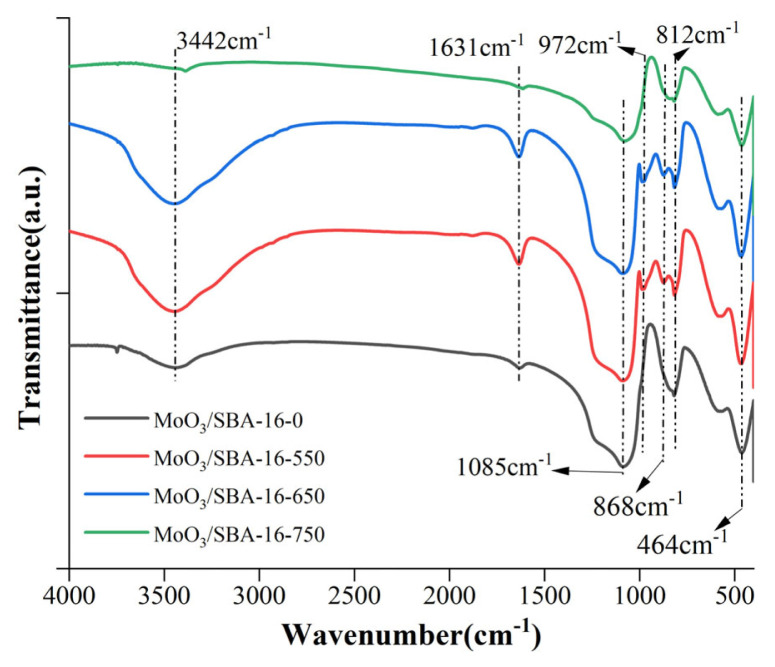
FT-IR image of MoO_3_/SBA-16 at different calcination temperatures.

It can be observed from the FT-IR (Figure 8) that the chemical bond composition of the MoO_3_/SBA-16 samples at different roasting temperatures. 812 cm^−1^ and 464 cm^−1^ bands are attributed to the formation of asymmetric stretching vibrations and bending stretching vibrations of the Mo-O-Mo single bond; 1085 cm^−1^ band is attributed to the symmetric stretching vibrations of Si-O-Si in the SiO_4_ tetrahedra [28]. The absorption peak at 962 cm^−1^ in the MoO_3_/SBA-16 sample is attributed to the stretching vibration of nonbridging oxygen atoms in Si-OH [29]. The strongest absorption peak at 972 cm^−1^ was observed for the modified catalyst at a calcination temperature of 550 °C, indicating that this temperature is more suitable for the interaction between MoO_3_ and SBA-16 during the hydrothermal process. The intensity of the absorption peak at 972 cm^−1^ gradually decreased with the increase of the firing temperature, indicating that some Si-OH groups on the surface of the mesoporous molecular sieve were consumed and converted into Si-O-Mo bonds, which further proved the incorporation of molybdenum species into the skeleton of SBA-16. This is in agreement with the XRD results.

**Figure 9:**
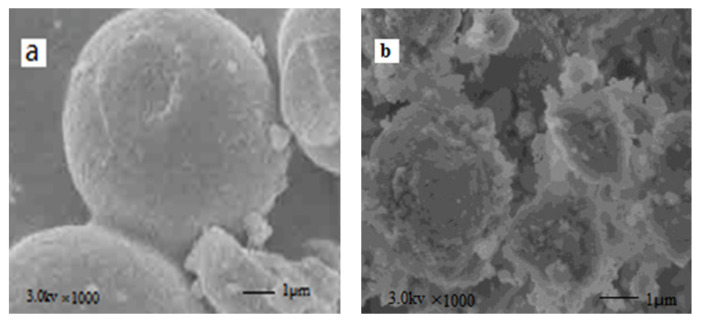
SEM spectra of SBA-16 and MoO_3_/SBA-16-550. a: SBA-16; b: MoO_3_/SBA-16-550.

**Figure 10:**
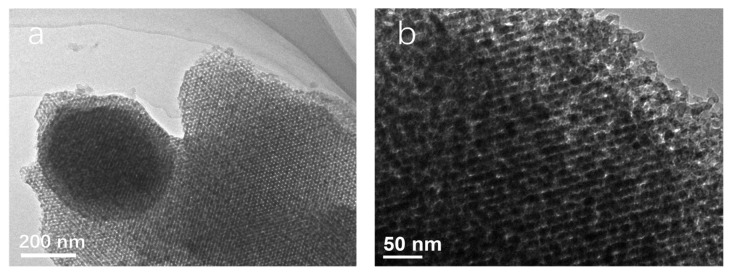
TEM images of MoO_3_/SBA-16-550 at different magnifications.

SEM images further verified the morphological structure of SBA-16 and MoO_3_/SBA-16 materials at 550 °C. The scanning electron microscope image in Figure 9a shows that SBA-16 material has a spherical structure with regular surface, and the particle size of the sample is about 2–4 μm. The surface is corrugated and has grooves, and the particles are evenly distributed. It is obvious that the particles accumulate to form a developed porous structure. On the one hand, it may be due to the selection of F127, which forms spherical micelles in the aqueous solution, as the template. On the other hand, the increase in seed crystal concentration promotes its further deposition on the surface of the polyhedron, so that the corners of the polyhedron disappear and become spherical. Scanning electron microscope image in Figure 9b shows that the particle morphology of MoO_3_/SBA-16 also presents a spherical structure, but the surface is relatively rough compared with SBA-16 particle morphology, and the particle size is about 3.0–5.0 μm, which is increased compared with SBA-16. It shows that part of molybdenum in ammonium molybdate does not directly enter the cage structure of SBA-16, but only exists on the surface of SBA-16. More interestingly, the diffraction peak intensity of the (110) crystal plane of the material gradually weakens and broadens, which may be due to the fact that the addition of molybdenum species weakens the electrostatic interaction, hydrogen bond and Van der Waals force between EO block and silicon cation, which affects the formation of SiO_2_ structure and causes some pores to collapse [30,31]. This confirms the analysis description of the XRD spectrum in Figure 2. The TEM image analysis in Figure 10 further illustrates that the SBA-16 in the MoO_3_/SBA-16 sample was composited by the nanoscale MoO_3_ grains. Even more importantly, it can be demonstrated from an enlarged view that these two components of SBA-16 and MoO_3_ are mixed with some interface between two materials, indicating the existence of composite interface.

## 4. Catalytic performance of catalyst

**Figure 11:**
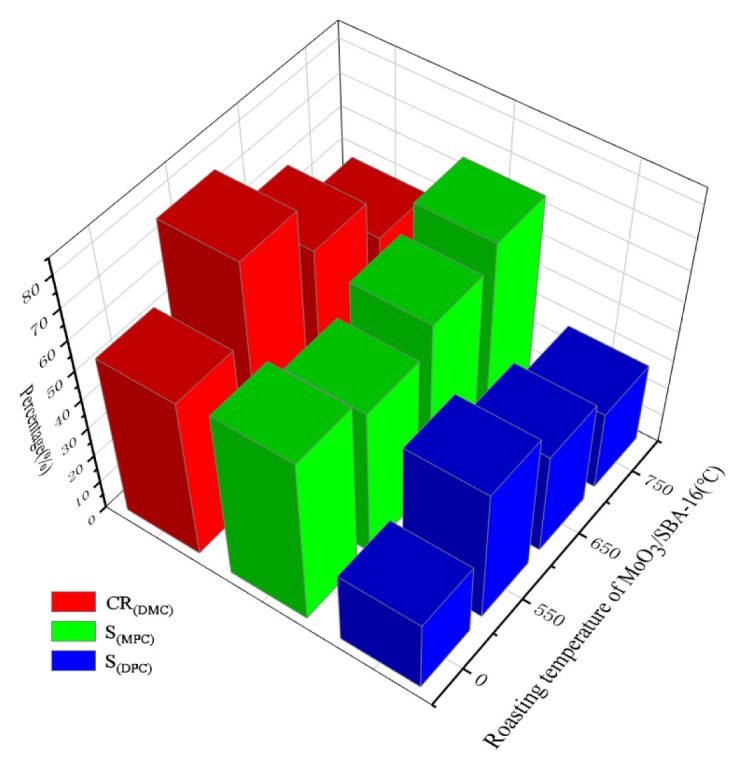
Catalytic performance of MoO_3_/SBA-16 at different calcination temperatures for transesterification of DMC and PA. Reaction conditions: n(PA) = 1.0mol; Si/Mo = 1.0; n(PA)/n(DMC) = 2.0; m(MoO_3_/SBA-16) = 6.0 g; t = 5 h; T = 180 °C. DMC: dimethyl carbonate; PA: phenyl acetate; MPC: methyl phenyl carbonate; DPC: diphenyl carbonate. Numbers 0 °C, 550 °C, 650 °C and 750 °C are calcination temperatures of the MoO_3_/SBA-16.

The MoO_3_/SBA-16 catalyst with different calcination temperatures was used to investigate the DMC conversion, selectivity and catalytic performance of methyl benzoate (MPC) and DPC in the synthesis of diphenyl carbonate (DPC) from dimethyl carbonate (DMC) and phenyl acetate (PA). The analysis of the results in Figure 11 shows that the prepared MoO_3_/SBA-16 catalyst shows relatively high catalytic performance; however, the performance of MoO_3_/SBA-16-0 without calcination is poor. The α-MoO_3_ catalyst at 50 °C shows relatively high catalytic performance, with DMC conversion of 78.5%, good stability and excellent catalytic performance in the reaction process. However, with the increase of temperature, the activity and catalytic performance of MoO_3_/SBA-16 catalyst decreased at 750 °C, and the conversion of DMC and the selectivity of DPC decreased, thus the stability of the reaction decreased. On the contrary, the selectivity of MPC increases. It is worth mentioning that MoO_3_/SBA-16-550, MoO_3_/SBA-16-650 and MoO_3_/SBA-16-750 catalysts are more selective to MPC than MoO_3_/SBA-16-0. It indicates that the addition of Mo species improved the silicon enrichment phenomenon on the surface of SBA-16, improved the dispersion and active sites, and enhanced the catalytic performance. Therefore, it is further demonstrated that MoO_3_/SBA-16 catalysts with different calcination temperatures have a little effect on the ratio of DMC to PA, but have a significant effect on the selectivity and stability of DMC and DPC.

Combined with the test analysis of XRD, TG-DTA, XPS, ICP-MS, FT-IR, BET, SEM, and TEM, it is shown that the calcination temperature conditions have an impact on the phase structure, morphology and specific surface area of MoO_3_/SBA-16 catalyst, and finally on its catalyst performance. According to the analysis, the calcination temperature of 550 °C for MoO_3_/SBA-16 was more favorable for the synthesis of DPC by transesterification of DMC and PA. Compared with previous researchers’ reports, the performance has been well optimized [32–34]. It is suggested that the addition of Mo species improved the silicon enrichment on the surface of SBA-16, increased the number of catalyst active sites, improved its dispersibility and accelerated the material transport.

## 5. Catalytic performance of transesterification of DMC and PA under different reaction conditions

The ratio of raw materials (n(PA)/n(DMC)) plays an important role in the transesterification reaction of DMC and PA. Therefore, first examine the ratio of raw materials (n(PA)/n(DMC)) to the transesterification reaction of DMC and PA. The catalytic performance. According to Table 2, the catalytic activity of MoO_3_/SBA-16 catalyst has a great relationship with the raw material ratio (n(PA)/n(DMC)). With the increase of n(PA)/n(DMC), the conversion of dimethyl carbonate and the selectivity of diphenyl carbonate increased, while the selectivity of methyl phenyl carbamate decreased gradually. The results indicated that the increase of PA dosage was beneficial to DMC conversion and DPC formation. When the ratio of raw materials n(PA)/n(DMC) = 5.0, the conversion rate of dimethyl carbonate reaches the maximum of 98.6%. It is worth mentioning that, with the further increase of the ratio of raw materials (n(PA)/n(DMC)), the selectivity of diphenyl carbonate will gradually increase, while the selectivity of methyl phenyl carbamate will still gradually decrease, but the conversion rate of dimethyl carbonate will start to decrease at an inflection point. This may be related to the fact that phenyl acetate accounts for a large proportion in the raw materials. Therefore, the ratio of n(PA)/n(DMC) = 2.0 is favorable for the transesterification of DMC and PA.

Table 3 is a study on the catalytic performance of MoO_3_/SBA-16 catalyst dosage for the transesterification of DMC and PA. It can be seen from Table 3 that with the increase of MoO_3_/SBA-16 catalyst dosage from 2.0 g to 8.0 g, the conversion rate of dimethyl carbonate and the selectivity of diphenyl carbonate in the transesterification of dimethyl carbonate and phenyl acetate show a gradual increase trend, while the selectivity of methyl carbamate decreases with the increase of MoO_3_/SBA-16 catalyst dosage. This is because with the increase of catalyst dosage, more active centers are produced, which is beneficial to the reaction. When the amount of catalyst is 6.0 g, the conversion of dimethyl carbonate and the selectivity of diphenyl carbonate are the highest, which are 78.5% and 46.1%, respectively. At the same time, compared with the dosage of single MoO_3_ catalyst (m = 6.0 g) [35,36], the effective dosage of active component MoO_3_ is reduced by nearly 81%, and the catalyst cost is greatly reduced. Further increasing the amount of MoO_3_/SBA-16 catalyst, the conversion rate of dimethyl carbonate and the selectivity of diphenyl carbonate did not increase, but the selectivity of methyl phenyl carbamate remained basically unchanged. This is mainly because excessive MoO_3_/SBA-16 catalyst reduces the amount of dimethyl carbonate which plays an effective role in transesterification, thus affecting the selectivity of diphenyl carbonate. Therefore, m(MoO_3_/SBA-16) = 6.0 g is considered as the most suitable amount of catalyst for diphenyl carbonate production.

Reaction time is also an important reaction parameter. Table 4 shows the catalytic performance of reaction time on transesterification of DMC and PA. The reaction was carried out at 180 °C. As shown in Table 4, prolonging the reaction time is beneficial to the conversion rate of dimethyl carbonate and the selectivity of diphenyl carbonate, while the selectivity of methyl phenyl carbamate gradually decreases. The possible reason is that MPC is transformed into DPC with the progress of the reaction. The possible reason is that MPC is transformed into DPC with the progress of the reaction. When the reaction time is increased to 5 h, the conversion rate of dimethyl carbonate and the selectivity of diphenyl carbonate are the highest, which are 78.2% and 45.9%, respectively. However, continuing to prolong the reaction time did not improve the conversion rate of dimethyl carbonate and the selectivity of diphenyl carbonate, and the selectivity of methyl carbamate remained basically unchanged, indicating that the transesterification reaction reached an equilibrium at this time. Therefore, the most suitable reaction time for transesterification of dimethyl carbonate with phenyl acetate is 5 h. According to the previous reports of researchers [35,36], the reaction time was shortened by nearly 30%, and the space-time yield of the catalyst was increased.

Because the reaction is influenced not only by kinetics, but also by chemical thermodynamics, the reaction temperature is one of the essential factors to explore the best catalytic conditions. With other parameters unchanged, the influence of temperature on the transesterification activity of DMC and PA was investigated in the temperature range of 150–200 °C As shown in Table 5, When the reaction temperature is 150 °C, the conversion of dimethyl carbonate and the selectivity of diphenyl carbonate are lower. When the temperature increases to 180 °C, the conversion rate of dimethyl carbonate and the selectivity of diphenyl carbonate increase to 78.5% and 46.5%, respectively, while the selectivity of methyl phenyl carbamate gradually decreases, which indicates that increasing the reaction temperature is beneficial to the transesterification of DMC and PA, because high temperature can better activate macromolecules and accelerate the transesterification to a high equilibrium constant [37]. Interestingly, when the reaction temperature is further increased, the conversion rate of dimethyl carbonate and the selectivity of diphenyl carbonate remain basically unchanged, while the selectivity of methyl carbamate increases. This is mainly because too high temperature will activate the transesterification reaction between DMC and PA, and promote the methyl carbamate reaction, which is no longer affected by temperature. Based on the above factors, 180 °C is considered as the best reaction temperature for transesterification of DMC and PA.

Table 6 shows the results of repeated use of MoO_3_/SBA-16-550 catalyst. It can be seen from the table that the activity of MoO_3_/SBA-16-550 catalyst decreased significantly after the 5th use. However, the performance of the regenerated catalyst was close to that of the first catalyst when the catalyst was calcined and regenerated at 550 °C under polite atmosphere for five times, indicating that the catalytic performance remained basically the same when the catalyst was calcined and regenerated for several times. Therefore, MoO_3_/SBA-16 can be used as a highly efficient catalyst for transesterification of DMC and PA.

**Figure 12:**
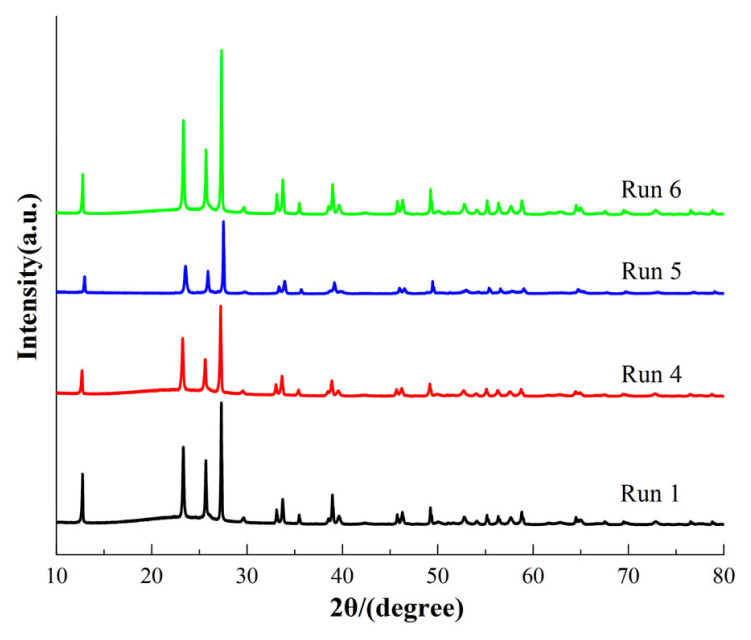
XRD patterns of the reused MoO_3_/SBA-16-550 catalyst.

Figure 12 shows the XRD patterns of the repeatedly used MoO_3_/SBA-16-550 catalysts. As can be seen from the figure, the crystal structures of the catalysts after four and five repetitions remained unchanged, but the diffraction peak signals of the catalysts with five repetitions decreased to some extent, and the intensity of the regenerated catalysts with five repetitions at 550 °C was similar to that of the catalysts with the first use, which, combined with the analysis in Table 4, indicates that the main reason for the decrease in catalytic performance may be related to the intensity of the catalyst diffraction peaks, further confirmed the Figure 2 XRD analysis.

## 6. Conclusion

In this part, SBA-16 molecular sieves with good thermal stability were synthesized by hydrothermal method under strongly acidic conditions, and MoO_3_/SBA-16 catalysts with high activity were prepared by room temperature impregnation strategy at 550 °C, 650 °C, and 750 °C. The effect of calcination temperature on the catalyst performance was investigated using uncalcined MoO_3_/SBA-16 catalysts as a reference. The structural activities of the catalysts were tested by XRD, TG-DTA, XPS, ICP-MS, FT-IR, BET, SEM, and TEM. It was found that the prepared MoO_3_/SBA-16-0, MoO_3_/SBA-16-550, MoO_3_/SBA-16-650 and MoO_3_/SBA-16-750 catalysts retained the structural characteristics of SBA-16, but the orderliness, specific surface area and catalytic performance of the MoO_3_/SBA-16 catalysts slightly decreased with the increase of calcination temperature. This proves that the calcination temperature is an important influencing factor for catalyst synthesis. It is worth mentioning that the addition of molybdenum species improved the silicon enrichment on the surface of SBA-16 molecular sieve and also increased the number of oxidation active sites of SBA-16. The MoO_3_/SBA-16 catalyst had the best catalytic performance in the esterification of DMC and PA when Si/Mo = 1.0 and calcination temperature was 550 °C. Under suitable reaction conditions (m(MoO_3_/SBA-16) = 6.0 g, n(PA) = 1.0 mol, n(PA)/n(DMC) = 2.0, T = 180 °C, t = 5 h), the conversion of DMC was 78.5%, and the selectivity of MPC and DPC was 51.1% and 46.5, respectively. More interestingly, the intensity of the diffraction peaks of the regenerated catalysts decreased after five repeated uses, but the crystal structure remained unchanged. The catalytic performance is close to that of the first use. This not only extends the lifetime of the catalyst but also reduces the production cost.

In summary, MoO_3_/SBA-16 has a high dispersibility and catalytic activity. It can be both reused many times and simply regenerated. As an efficient catalyst for the esterification of DMC and PA to DPC, it is suitable for industrial applications.

## Figures and Tables

**Table 1:** Structural characteristics of MoO_3_/SBA-16 catalysts at different calcination temperatures.

Sample	S_BET_ (m^2^ g^−1^)	V_total_ (cm^3^ g^−1^)	V_meso_ (cm^3^ g^−1^)	BJH pore size (nm)	V_meso_/V_total_ (%)
SBA-16	807.6134	0.853419	0.800553	7.52	93.81
MoO_3_/SBA-16-0	131.8974	0.604164	0.597552	12.58	98.91
MoO_3_/SBA-16-550	279.8093	0.575363	0.574199	6.41	99.80
MoO_3_/SBA-16-650	153.2075	0.488223	0.488076	7.56	99.97
MoO_3_/SBA-16-750	132.8715	0.278751	0.276694	6.37	99.26

V_total_: Total pore volume at P/P_0_ approximately 0.98.

V_meso_: Total pore volume subtraction micropore volume calculated by the t-plot method.

**Table 2:** Effect of n(PA)/n(DMC) on catalytic esterification of DMC and PA over MoO_3_/SBA-16.

n(PA)/n(DMC)	x(DMC)/%	s/%			
		MPC	DPC	MPC+DPC	PhOH
0.5	44.6	60.0	30.0	90.0	10.0
1.0	69.8	58.1	37.0	95.1	4.9
2.0	78.5	51.6	46.0	97.6	2.4
3.0	80.7	50.8	46.6	97.4	2.6
4.0	85.5	50.0	47.2	97.2	2.8
5.0	98.6	49.5	48.1	97.6	2.4
6.0	98.3	47.0	50.5	97.5	2.5

Reaction conditions: n(PA) = 1.0mol; Si/Mo = 1.0; t = 5.0 h; m(MoO_3_/SBA-16) = 6.0 g; T = 180 °C; DMC: dimethyl carbonate; PA: phenyl acetate; MPC: methyl phenyl carbonate; DPC: diphenyl carbonate. Calcination temperatures of the MoO_3_/SBA-16 is 550.

**Table 3:** Effect of m(MoO_3_/SBA-16) on catalytic esterification of DMC and PA over MoO_3_/SBA-16.

m(MoO_3_/SBA-16)	x(DMC)/%	s/%			
		MPC	DPC	MPC+DPC	PhOH
1.0	0	0	0	0	0
2.0	28.5	65.5	24.3	89.8	10.2
3.0	44.3	63.8	26.6	90.4	9.6
4.0	63.0	62.5	31.1	93.6	6.4
5.0	74.4	59.1	37.5	96.6	3.4
6.0	78.5	51.5	46.1	97.6	2.4
7.0	78.0	51.8	45.6	97.4	2.6
8.0	76.1	53.1	44.0	97.1	2.9

Reaction conditions: n(PA) = 1.0 mol; Si/Mo = 1.0; n(PA)/n(DMC) = 2.0; t = 5.0 h; T = 180 °C; DMC: dimethyl carbonate; PA: phenyl acetate; MPC: methyl phenyl carbonate; DPC: diphenyl carbonate. Calcination temperatures of the MoO_3_/SBA-16 is 550.

**Table 4:** Effect of reaction time on catalytic esterification of DMC and PA over MoO_3_/SBA-16.

t/h	x(DMC)/%	s/%			
		MPC	DPC	MPC+DPC	PhOH
1.0	33.2	71.5	21.3	92.8	7.2
2.0	56.5	68.1	25.8	93.9	6.1
3.0	65.2	67.1	28.2	95.3	4.7
4.0	71.1	60.9	35.6	96.5	3.5
5.0	78.2	51.6	45.9	97.5	2.5
6.0	78.3	51.8	45.5	97.3	2.7

Reaction conditions: n(PA) = 1.0 mol; Si/Mo = 1.0; n(PA)/n(DMC) = 2.0; m(MoO_3_/SBA-16) = 6.0 g; T = 180 °C; DMC: dimethyl carbonate; PA: phenyl acetate; MPC: methyl phenyl carbonate; DPC: diphenyl carbonate. Calcination temperatures of the MoO_3_/SBA-16 is 550.

**Table 5:** Effect of reaction temperature on catalytic esterification of DMC and PA over MoO_3_/SBA-16.

T/°C	x(DMC)/%	s/%			
		MPC	DPC	MPC+DPC	PhOH
150	0	0	0	0	0
160	67.4	64.6	30.1	94.7	5.3
170	73.9	58.9	36.5	95.4	4.6
180	78.5	51.1	46.5	97.6	2.4
190	78.7	52.3	45.1	97.4	2.6
200	78.1	52.2	45.1	97.3	2.7

Reaction conditions: n(PA) = 1.0mol; Si/Mo = 1.0; n(PA)/n(DMC) = 2.0; m(MoO_3_/SBA-16) = 6.0 g; t = 5.0 h; DMC: dimethyl carbonate; PA: phenyl acetate; MPC: methyl phenyl carbonate; DPC: diphenyl carbonate. Calcination temperatures of the MoO_3_/SBA-16 is 550.

**Table 6:** Reuse of the MoO_3_/SBA-16-550 catalyst.

Run	x(DMC)/%	s/%			
		MPC	DPC	MPC+DPC	PhOH
1	78.5	51.6	46.0	97.6	2.4
2	78.3	51.0	46.3	97.3	2.7
3	76.9	51.0	45.5	96.4	3.5
4	69.7	54.3	42.8	97.1	2.9
5	15.8	54.5	42.4	96.9	3.1
6	77.2	50.7	46.4	97.1	2.9

Reaction conditions: n(PA) = 1.0 mol; Si/Mo = 1.0; t = 5.0 h; m(MoO_3_/SBA-16) = 6.0 g; T = 180 °C. Run 6 was generated by calcining the sample used for run 5 at 550 °C for 5 h.
